# Epidemiology and utilization of primary health care services in Qatar by asthmatic children 5–12 years old: secondary data analysis 2016–2017

**DOI:** 10.1186/s40733-019-0050-4

**Published:** 2019-08-20

**Authors:** Shajitha Thekke Veettil, Ahmed Sameer AbdulHameed Alnuaimi

**Affiliations:** 1 Clinical Research Department, Primary Health Care Corporation, Head Quarters Tower 1, 8th Floor, PO Box: 26555, Doha, Qatar; 2 Directorate of Clinical Affairs, Al Salata, Al Meena Street, Doha, Qatar

**Keywords:** Childhood asthma, Primary health care, Utilization, Seasonal variation, Pharmacotherapy, Qatar

## Abstract

**Background:**

Childhood asthma is a growing clinical problem and a burden on the health care system due to repetitive visits to children’s emergency departments and frequent hospital admissions where it is poorly controlled. Due to lack of reliable baseline information on its prevalence among children in Qatar and the extent of their utilization of primary health care services, we sought to analyse electronic medical records data for children aged 5–12 years.

**Objectives:**

Our primary objective was to establish point prevalence over the period 2016–2017. Furthermore, we wanted to assess the frequency and pattern of use of the primary care services including any demographic and seasonal variations, the types of clinical encounter and treatment received.

**Methods:**

A cross sectional study on 54,704 clinical encounters of electronic health records for children aged 5 to 12 years in which a diagnosis of Asthma was tagged during a two years period.

**Results:**

The prevalence rate of Asthma out of total registered clients in the specified pediatric age group (196,557) is 6.1%. The rate was highest (10.2%) in youngest age group (5–6 years old) and lowest (4.1%) in teenagers (10–12 years old). An obvious peak of clinical encounters of Asthma cases was observed in Oct and Nov. The work load in PHCC clinics for Asthma clinical encounters is increased by more than 50% compared to the average monthly count of 4556.Moreover, the rate was higher in males (7.6%) compared to females (4.6%). The most frequently prescribed medication group was antihistamine (57.8%) followed by adrenergic bronchodilators (33.9%).

**Conclusions:**

Asthma constitutes an important part (8.5%) of the total primary care clinic work load among children aged 5–12 years in Qatar. A guideline need to encourage physician to use preventive Asthma strategies including steroid medications to provide continuity of care for Asthma cases.

## Background

Bronchial asthma is one of the most common chronic diseases with a varying age of onset, affecting all age groups. Whilst recent reports indicate an increasing prevalence especially among children, the accuracy of reporting is difficult to ascertain. In 2014, the Global Asthma Report [1] estimated that 334 million people have asthma. The report also projected an additional 100 million new cases by 2025. Early detection and improved health care have resulted in a decline in hospitalizations and deaths from childhood asthma in some countries, yet the burden on health care systems [1–3] and economic costs to society through loss of productivity in the workplace and disruption to family life remain unacceptably high [1].

In some industrialized countries, asthma and related allergies affect more than one-third of the child population [4, 5] although it remains unclear whether these are real term increases in prevalence, the result of a higher level of awareness and better detection rates [4, 6] or a combination of the two. In the Middle East and Arab Gulf countries with rapidly expanding industrial economies over the last three decades, prevalence rates are estimated to be lower than most Organisation for Economic Co-operation and Development (OECD) countries. For example, in a recent systematic review, mean estimates of asthma in school-age children were reported to be 7.6% (95% Cl: 6.4–8.8) in the Middle East region, but varied from 0.7% in Isfahan, Iran to 19.8% (Qatar), 20.7% (Oman) and 22.3% in Iraq [6]. In comparison, prevalence rates of 12.8% (Spain), 17.8% (Turkey), 25.9% (United Kingdom) and 31% (Australia) have been reported [7]. On the contrary, lower rates have been reported in the Tibet area in China (1.1%), India (4.9%) and Taiwan (6%) with a male preponderance although there appears to be a convergence between the sexes in early adolescence [5].

Of particular concern is the importance of reliable baseline information on the prevalence of childhood asthma in relation to their utilization of primary health care services for routine follow-up and acute care of mild disease, rather than secondary hospital services. In Qatar, it has been reported that up to 5000 children with asthma visit the Chest Clinic at secondary care hospitals for treatment and follow up appointments annually [7] although this secondary care data provided was also reported to be incomplete. However, more importantly what is lacking is consistent, reliable and longitudinal primary care data to allow more accurate reporting and assessment of trends over time.

As a chronic disease, the emphasis on its effective management should focus on achieving clinical control, preventing future risk of exacerbations and ensuring patient and family education on how to minimize exposure to triggers of acute attacks and what to do when such acute episodes do occur. Strategies which result in well-controlled asthma are associated with a significant reduction the economic burden of childhood asthma resulting from direct healthcare costs, and indirect costs [1] including pharmacotherapy.

### Rationale of study

Frequent pediatric emergency department visits and hospitalizations due to acute asthma and frequent absences from school have consequences for the individual child, family and their performance [8–10]. Quantifying the size of the problem and planning effective preventive interventions require adequate incidence and prevalence data, and the frequency and types of clinical encounters and their management including the pharmacological treatment of children with asthma. A recent clinical audit of the use of clinical guidelines for childhood asthma management in primary care in Qatar have recommended the need for strict adherence to the Qatar Ministry of Public Health recommended national Global Initiative for Asthma GINA framework [11, 12]. More recently an electronic medical records (EMR) system has been established for recording and tracking all clinical encounters in primary care in Qatar.

### Aim of study

Our primary objective was to analyze existing EMR data to determine prevalence rate and utilization patterns of primary health care services in Qatar by children diagnosed with asthma in the age group 5–12 years over the 2016 and 2017 period.

### Objectives

1. Assess the association between seasonal variation, age and gender and the percentage of Asthmatic clinical encounters out of total PHCC clinic workload.
2. Calculate the prevalence rate of Asthma out of total registered clients and assess the association with age and gender.
3. Assess the utilization pattern of PHCC health services to pediatric cases with Asthma by measuring the count of visits during the 2 years study period and the median interval (days) between successive visits.
4. Describe the pharmacotherapy for Asthma cases during the study period.

## Material and methods

### Study design

Cross sectional secondary data analysis of electronic medical records.

### Study setting

The Primary Health Care Corporation (PHCC) is the governmental organization of Qatar responsible for the delivery of primary health care services to population of Qatar. Although the State of Qatar took its first steps in establishing a primary health care system and started to provide healthcare services through a range of clinics as early as 1954, the PHCC was established as an independent corporation with its own independent budget in 2012. By the end of 2017, the Primary Health Care Corporation was operating through 23 primary health care. Thirteen of these centers are located in Doha city, while the rest of centers will be located in populated areas in all parts of Qatar. The two health systems of Qatar HMC (Hamad Medical Corporation) and PHCC together serve more than 90% of the country’s population of 2.3 million people.

The electronic medical records (CERNER) was introduced in PHCC health centers from the 1st of January 2016.

### Study population

The study population consists of all registered cases having at least one visit (clinical encounter) in any of the 23 clinics operated by PHCC with a verified diagnosis of Asthma. Only children aged 5 to 12 years old during the period 1st Jan 2016 to 31st Dec 2017 were eligible for inclusion.

### Study sample

No sampling is required, since all the population is analyzed.

### Data management

A total of 54,704 clinical encounters that satisfied the inclusion criteria were retrieved from the CERNER system. These encounters had Asthma tagged as a chronic condition. These records were aggregated to 12,064 unique subjects with a diagnosis of Asthma during the study period. The count of total registered subjects in the specific age group by the end of 2017 (196,557) was stratified by age and gender to help in calculating prevalence rates.

An estimate of prevalence during the two years study period was calculated by dividing the count of cases during the study period by the total registered clients of the same age group in PHCC health centers by the end of 2017. No attempt was made to break the results by year to reduce the differences attributed to unstable data (the electronic system started in 2016 and needed some time to settle). Whatever differences exist between the two years is attributed to administrative issues and does not reflect differences between two years.

### Data analysis

SPSS (Statistical Package for Social Sciences V. 23) was used for statistical analysis of data. Frequency distribution and appropriate graphical presentations were done. No analytic statistical analysis or test of significance was required, since all the population was analyzed.

## Results

The results presented in this chapter were based on the analysis of 54,704 clinical encounters in which Asthma is tagged in the electronic record as a chronic condition.

**Fig. 1:**
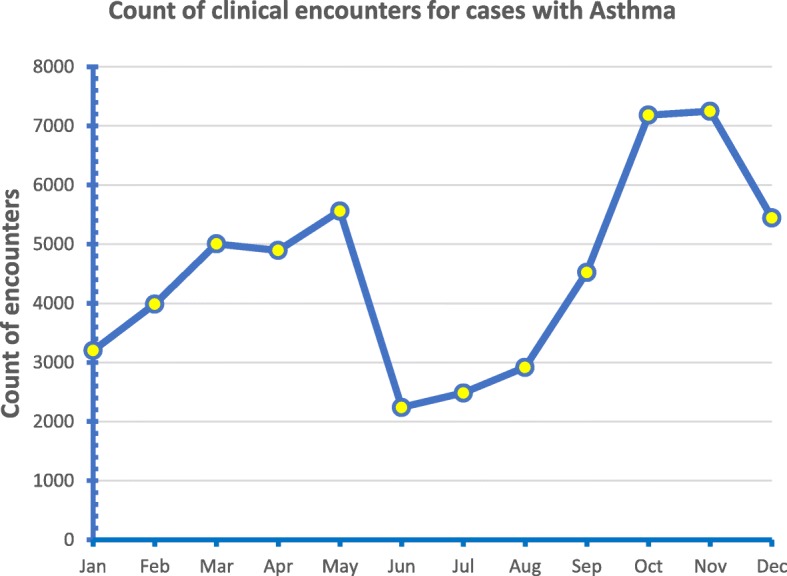
Line graph showing the monthly time trend for the count of clinical encounters with a tag of Asthma as a chronic condition during the study period

As shown in Fig. 1, an obvious peak of clinical encounters of Asthma cases was observed in Oct and Nov. The work load in PHCC clinics for Asthma clinical encounters is increased by more than 50% compared to the average monthly count of 4556.

**Fig. 2:**
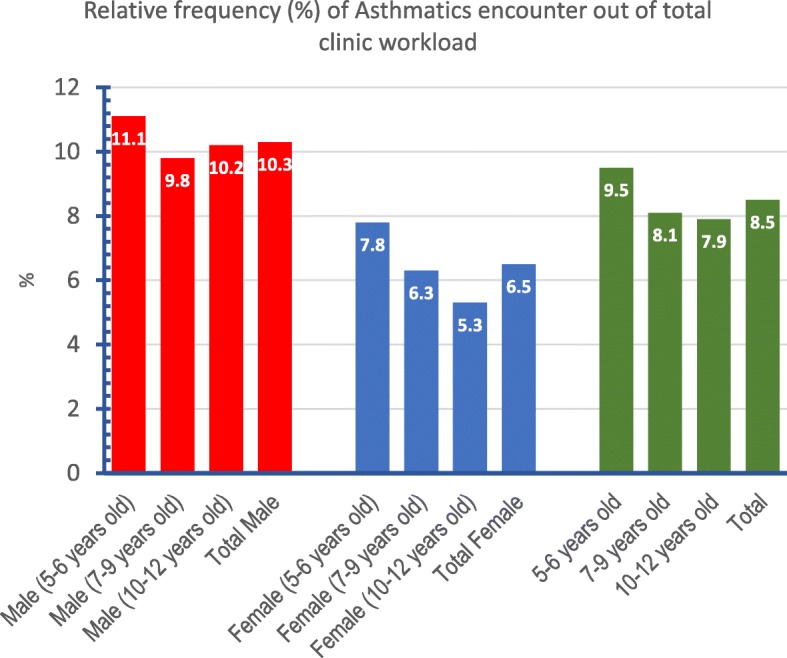
Bar graph showing the percentage of clinical encounters with a tag of Asthma as a chronic condition out of total clinical encounters (with any complaint or reason for visit) stratified by age and gender

**Fig. 3:**
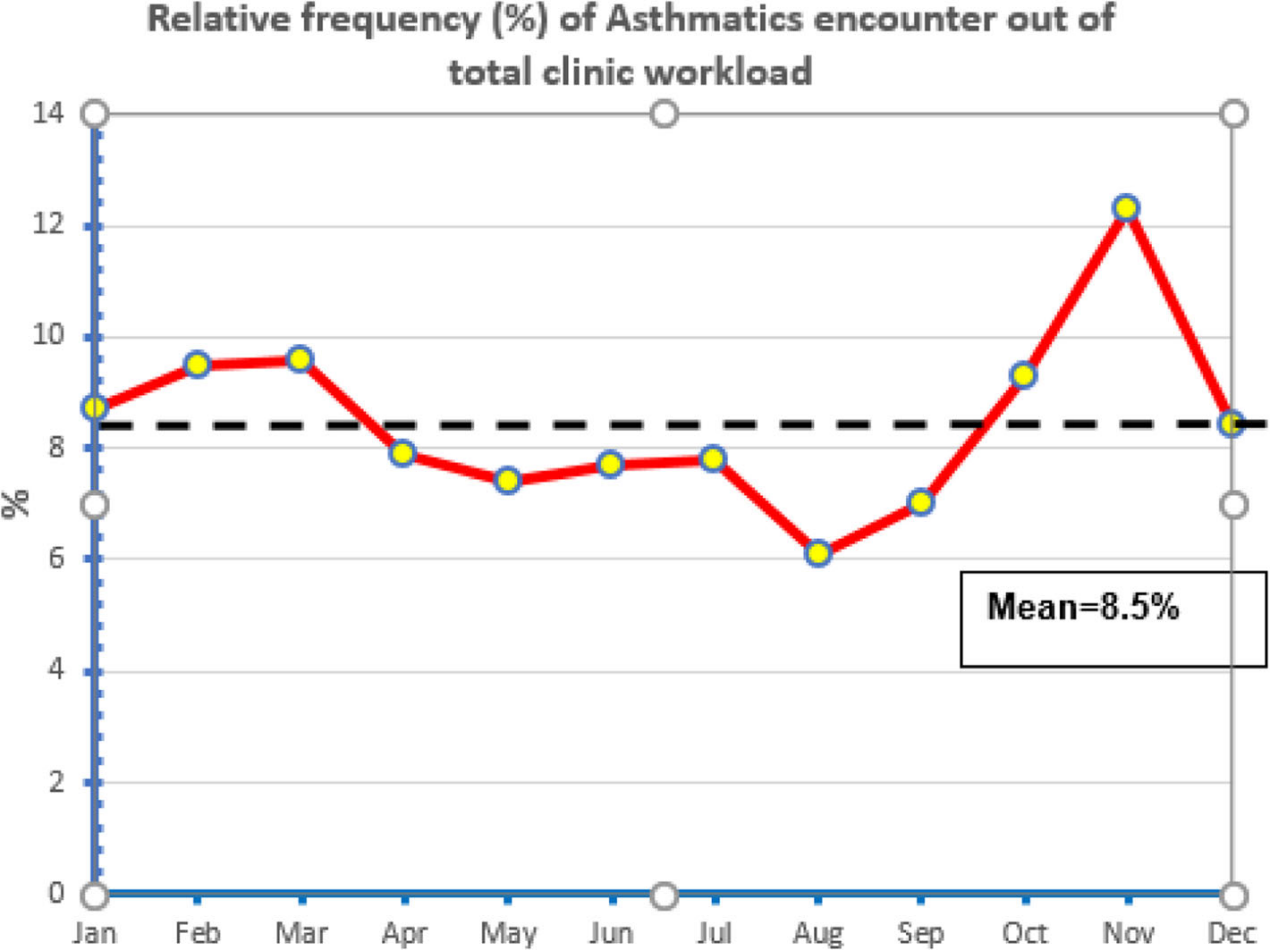
Line graph showing the monthly time trend for the percentage clinical encounters with a tag of Asthma as a chronic condition out of total clinical encounters (with any complaint or reason for visit) during the study period

As shown in Fig. 2, the percentage of Asthmatic clinical encounters out of total PHCC clinic workload was 8.5%. This rate was higher (9.5%) for younger age groups (5–6 years old). In addition, it was higher in males (10.3%) compared to females (6.5%). November month was associated with an obvious increase in this rate (12.3%) compared to the average monthly workload of 8.5%, Fig. 3.

**Fig. 4:**
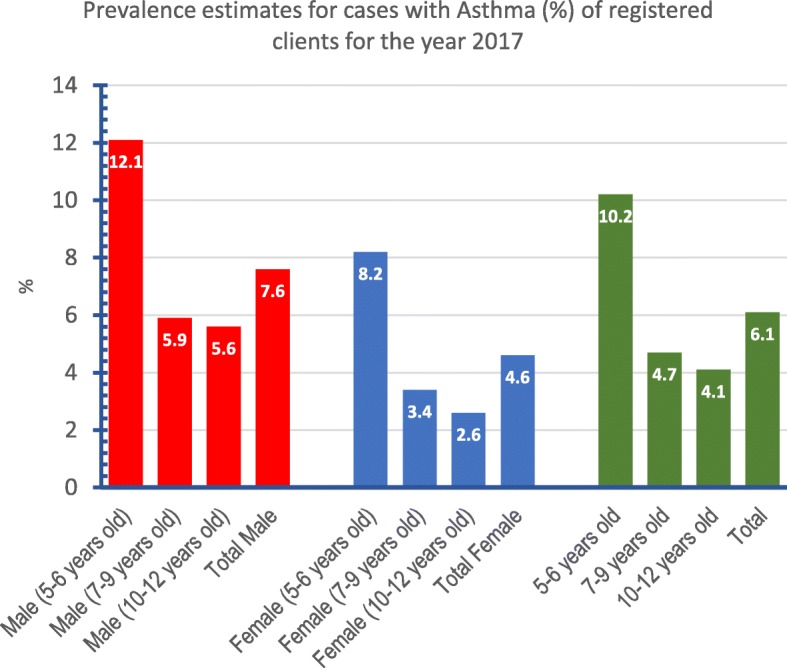
Line graph showing the monthly time trend for the estimated prevalence rate of Asthma stratified by age and gender

As shown in Fig. 4, the prevalence rate of Asthma out of total registered clients in the specified pediatric age group (196,557) is 6.1%. The rate was highest (10.2%) in youngest age group (5–6 years old) and lowest (4.1%) in teenagers (10–12 years old). Moreover, the rate was higher in males (7.6%) compared to females (4.6%).

As shown in Table 1, about a quarter (27.1%) of total registered cases with Asthma (*n* = 12,064) in the specified age group had only a single visit, while those with more than 5 visits constituted 27.8%.

**Table 1:** Frequency distribution of registered cases with Asthma by count of visits during the 2 years study period

	N	%
Count of visits-categories		
Single visit	3271	27.1
2–5	5448	45.2
6–9	1951	16.2
10+	1394	11.6
Total	12,064	100

In addition, those with a short average duration between successive visits (4 weeks or shorter) constituted more than a quarter (27.7%) of total registered cases with Asthma, Table 2.

**Table 2:** Frequency distribution of registered cases with Asthma by median interval (days) between successive visits

	N	%
Median interval (days) between successive visits-categories		
< 2 weeks	1035	11.8
2–4 weeks	1389	15.9
> 4 weeks- < 6 months	5130	58.6
6 months +	1197	13.7
Total	8751	100.0

**Table 3:** Frequency distribution of registered cases with Asthma by categories of drugs prescribed at least once

At least once prescribed (Total *N* = 12,064)	N	%
Anti-histamine	6972	57.8
Adrenergic bronchodilators	4088	33.9
Steroid medications	2742	22.7
Leukotriene receptor antagonist	903	7.5

As shown in Tables 3 and 4, five categories of pharmacotherapy were considered and assessed for being used at least once by an Asthmatic during the study period. The most frequently prescribed medication group was antihistamine (57.8%) followed by adrenergic bronchodilators (33.9%). Steroids were prescribed for only 22.7% of cases, while Leukotriene receptor antagonists were used in 7.5% of cases only. The use of steroid medications was very low among single visit Asthmatics (9.2%) and increases to a maximum of 75% among those with very frequent clinic visits (> 10 times during two years).

**Table 4:** The relative frequency of prescribing selected categories of drugs at least once for registered cases with Asthma by count of clinical encounters (visits) during the two years study period

	Count of visits-categories									
	Single visit		2–5		6–9		10+		Total	
At least once prescribed	N (total *n* = 3271)	%	N (Total *n* = 5448)	%	N (Total *n* = 1951)	%	N (Total *n* = 1394)	%	N (total *n* = 12,064)	%
Anti-histamine	186	5.7	1022	18.8	715	36.6	819	58.8	2742	22.7
Adrenergic bronchodilators	734	22.4	3132	57.5	1745	89.4	1361	97.6	6972	57.8
Steroid medications	302	9.2	1654	30.4	1087	55.7	1045	75.0	4088	33.9
Leukotriene receptor antagonist	61	1.9	328	6.0	236	12.1	278	19.9	903	7.5

## Discussion

The implementation of electronic medical recording system (CERNER) in primary health care setting in Qatar starting 2016 provided the opportunity to study the utilization of these services and provide some epidemiologic insights into one of the important health problems in pediatrics.

In this cross sectional study, electronic health records for children aged 5 to 12 years in which a diagnosis of Asthma was tagged were extracted during a two years period (2016–2017). The seasonal pattern was analyzed after aggregating the two years data to reduce the random variation component between the two years. The peak of clinical encounters of asthma cases increased by Oct and Nov more than 50% compared to the average monthly count. This pattern was endorsed by analyzing the encounters as a proportion out of total clinic workload for the same months. In November the contribution of Asthma clinical encounters to total clinic workload is increased by an almost a half compared to the monthly average of 8.5%. The seasonal pattern for Asthma has important impact on the health care system. Hospital admissions as well as emergency department (ED) visits for asthma exacerbations would differ among school-age children who lived in locations with different climates and these differences have important therapeutic implications [13].

Seasonal variations in the frequency of asthma exacerbations during childhood occur worldwide. Among preschool and older children, most of the seasonal information available has been derived from studies of children who lived in the United States, Canada, the United Kingdom, and northern Europe [14, 15]. These studies reported an increase in wheezing attacks that are most pronounced during the fall months. Some studies also reported an increase in exacerbations in the spring [14, 16]. It is not clear, however, whether seasonal patterns for attacks of wheezing are the same among children who live in different geographic regions where climates and environmental conditions vary. Several articles proposed that viral infections account for the increased frequency of asthma exacerbations in the fall when children return to school [17–20]. Other studies indicate that exposure to environmental allergens, which vary in intensity at different times of the year may provide an explanation for this peak [14, 15]. Huang S-J, et al., concluded in their study that 70% of the children with asthma in Taiwan had airway hyper-responsiveness methacholine, which varied among seasons. Children with a higher total serum IgE level may be more seasonally dependent, particularly in summer [21]. A frequency of attacks was observed during the summer months in all the locations. Seasonal peaks for asthma exacerbations varied among the children who lived in geographic locations with different climates, and were not restricted to the beginning of the school year [13].

Environmental pollution may also contributed to seasonal variation of Asthma clinical encounters. According to the article from Doha news, the capital of Qatar “Doha” is ranked by the World Health Organization among the world’s most polluted cities. Unfortunately, official Air Quality monitoring in Qatar is not available. But from this article, the Qatar Environment and Energy Research Institute is working, since 2013, on setting up the official Air Quality Monitoring System in Doha and still waiting for the official data to be published [22].

A school-based asthma trial conducted by the University of Rochester School of Medicine and Dentistry, Rochester, NY, USA recommend that children with persistent asthma have at least 2 preventive asthma visits per year [23]. Analysis of Asthma encounters in PHCC showed around a quarter of cases achieved only one visit to the health center during the whole study period. This may point out to a possible wrong diagnosis, since no pulmonary function test is done to establish the diagnosis in PHCC clinics. The GINA guidelines [24] clearly require the pulmonary function test to establish the diagnosis. Another possibility is a perceived low satisfaction from the patient or his parents with the health care service or its timing. Slightly more than a quarter of Asthmatic cases visited the clinics more than 5 times during the two years period, which obviously exceed the recommended frequency of visits per year and places an added burden on the clinical staff workload. Some of these asthmatics had more than 10 consultations (clinical encounters) during the study period. In addition, those with a short average duration between successive visits (4 weeks or shorter) constituted more than a quarter (27.7%) of total registered cases with Asthma. Such a frequent visit and / or short duration between successive visits may be attributed to inadequate therapeutic control or preventive strategy for Asthma. Non adherence to recommended clinical practice guidelines by physicians or lack of compliance with prescribed medications from patients are probable causes behind these findings.

An estimate for the age specific prevalence rate of Asthma during 2016–2017 was based on knowing the count of registered Asthma cases in a specific age group divided by the count of registered clients in the same age group by the end of 2017. This approach may suffer from a non-measurable amount and direction of bias, since the population of Qatar is a dynamic one with a noticeable amount of expatriates coming in and leaving out the country constantly. In addition, the private sector and secondary health care providers attract some of Asthma cases especially in the pediatric ages. Nevertheless having an estimate is better than guessing. The prevalence rate of asthma in PHCC pediatric clients was 6.1%. This rate was higher (10.2%) in younger children (5–6 years old) and decrease with advancing age to reach its lower value of 4.1% in teenagers (10–12 years old). More over the rate is higher in boys (7.6%) compared to girls (4.6%). These findings were previously reflected in a cross sectional study in Qatar in 2004 [6] . Other studies from the Middle East showed that the prevalence of asthma is lower than many developed countries such as the UK (25.9%), Spain (12.8%), Australia (31%) and Turkey (17.8%). However, it is higher than some developing countries like Tibet area in China (1.1%), India (4.9%) and Taiwan (6%). In addition, studies in the Middle East showed that the prevalence of asthma is higher among younger boys. However, the difference decreases between the two sexes in early adolescence [5] . This male gender predilection to Asthma was raised in other studies from Japan, Turkey, Nigeria, South Korea, and India [5].

In a systematic review Pearce et al. reported that the negative age trend for the prevalence of Asthma shown in the current study was also reported in 5 countries including Iran and Oman [25]. In that paper, they reported that across 35 countries of the world, the prevalence of asthma in 21 countries (60%) is higher in the older age group and in nine countries (25.7%) is higher in the younger age group. It was reported in 10 countries (25.7%) that the difference was insignificant. Overall, in the Middle East, the prevalence of asthma is higher in the 13–14 years age group [5].

The 2016 GINA report was intended to provide an advice on diagnosis and treatment of asthma and make it more personalized and responsive to individual patient’s needs. The “new” definition of asthma was suggested to describe its heterogeneous nature. The report emphasized the importance of confirming the diagnosis of asthma using lung function test to minimize both under and over-treatment. In addition, it highlighted a comprehensive approach to asthma management that acknowledges the foundational role of inhaled corticosteroid therapy. The framework for individualizing patient care addressed common problems such as incorrect inhaler technique and poor adherence; a continuum of care for worsening asthma, starting with early self-management and progressing to acute care management [26].

The use of steroids in management of Asthma was recorded at least once in only 22.7% of cases in the current study. This percentage is very low, especially if we consider that management of asthma patients is complicated by non-adherence to inhaled corticosteroid (ICS) therapy as a factor for increased exacerbation risk and therefore as a reason for more frequent visits to the health centers [27]. Referring to the GINA advice on using steroids, the current study showed that physicians in primary care are resistant to the idea of using preventive steroids medication for cases with Asthma. Only those with very frequent visits (> 5 per year) would be deemed eligible for using steroid by their managing physician. It is interesting to observe that even for those cases with very frequent visits, still a quarter had never used a steroid medication.

### Study limitations

It is expected that Asthma is over diagnosed in the present study, since pulmonary function tests are not available in primary health care setting. In addition, the calculation of prevalence rate may suffer from bias because using the registered cases as the population at risk is not an accurate representation for catchment area and some of the Asthma cases prefer to receive care in hospitals.

## Conclusion

1. The estimated prevalence of Asthma during 2016–2017 in registered PHCC clients of 196,557 is 6.1%.
2. The prevalence of Asthma is higher in males and in the younger age group of 5–6 years (which is almost double that in older age group of 10–12 years old).
3. Clinical encounters with a diagnosis of Asthma constitutes an important part (8.5%) of the total primary health care clinic work load in Qatar.
4. More than a quarter of cases labeled with Asthma have frequent visits to the clinics (> the recommended two visits per year).
5. The peak of Asthma cases is in Autumn Season with a distinguished peak in Nov.
6. Steroids are underutilized for preventing asthmatic attacks in primary health care settings.

### Recommendation

A guideline is needed to encourage physician to use preventive Asthma strategies including steroid medications. CERNER data can help in the continuity of care for Asthma cases guiding physicians in management of those with recurrent visits or those who are lost to follow up after a single visit.

## Data Availability

Not applicable

## Abbreviations

CERNER: Electronic Medical Records
EMR: Electronic Medical Records
GINA: Global Initiative for Asthma
OECD: Organisation for Economic Co-operation and Development
PHCC: Primary Health care Corporation
